# Urinary NGAL, IGFBP-7, and TIMP-2: novel biomarkers to predict contrast medium-induced acute kidney injury in children

**DOI:** 10.1080/0886022X.2022.2075277

**Published:** 2022-09-19

**Authors:** Qianliang Sun, Zhijuan Kang, Zhihui Li, Mai Xun

**Affiliations:** College of Pediatrics, University of South China/Children’s Hospital of Hunan Province, Changsha, P.R. China

**Keywords:** Acute kidney injury, biomarkers, contrast medium, child, urinary

## Abstract

**Background:**

Serum creatinine (SCr) is unreliable in detecting acute changes in kidney function. Early recognition of contrast-induced acute kidney injury (CI-AKI) can provide better opportunities for preventive interventions. Therefore, the purpose of this study is to examine the value of the combined detection of urinary neutrophil gelatinase-associated lipocalin (NGAL), insulin-like growth factor binding protein-7 (IGFBP-7), and tissue inhibitor of metalloproteinase-2 (TIMP-2) in the early diagnosis of children with CI-AKI.

**Methods:**

A prospective, single-center clinical trial was performed and included 172 children aged 0–18 years. The dynamic changes of urinary NGAL, IGFBP-7, and TIMP-2 levels in children with intravascular injection of contrast medium were investigated to determine whether they can diagnose CI-AKI early.

**Results:**

CI-AKI occurred in 20 of 137 enrolled patients, and the incidence was 14.59%. In the CI-AKI group, urinary levels of NGAL, IGFBP-7, TIMP-2, and [IGFBP-7]*[TIMP-2] were significantly increased 2 h after angiography and remained at high levels at 6 h. Using a cutoff value of 36.274 ng/mL, the specificity was 70.0%, and the sensitivity was 68.4% for the prediction of CI-AKI, which was excellent for urinary NGAL. When both urinary IGFBP-7 and TIMP-2 were used together, urinary [IGFBP-7]*[TIMP-2] at 0.417(ng/mL)^2^/1000 was regarded as the cutoff value. The specificity was 80.0%, and the sensitivity was 81.2%.

**Conclusions:**

NGAL, IGFBP-7, and TIMP-2 concentrations in the urine of children after receiving injections of contrast medium increased faster than SCr and had good clinical value for the early diagnosis of CI-AKI in children. The combination of IGFBP-7 and TIMP-2 was better than either analyte alone.

## 1. Introduction

Contrast-induced acute kidney injury (CI-AKI) refers to a decrease in renal function after intravascular injection of contrast medium (CM). In inpatient settings, CI-AKI is one of the most common iatrogenic causes of acute renal failure. With technological advances in diagnostic and interventional imaging technology, CI-AKI has become the third leading cause of acute kidney injury (AKI) in hospitalized patients [1,2]. The diagnosis of AKI depends on the increase in serum creatinine (SCr) levels. Unfortunately, creatinine is an unreliable indicator of acute changes in kidney function. This causes a delay in the diagnosis of CI-AKI, which affects treatment and corrective interventions [3–7]. Hence, early biomarkers of CI-AKI are crucial to improve the prognosis of patients with renal damage.

Several biomarkers have been investigated for the early diagnosis of CI-AKI [8–13], such as neutrophil gelatinase-associated lipocalin (NGAL), insulin-like growth factor-binding protein-7 (IGFBP-7), and tissue inhibitor of metalloproteinase-2 (TIMP-2). The renal damage caused by contrast agents cannot be ignored. Therefore, we conducted a prospective study to investigate the value of urinary NGAL, IGFBP-7, and TIMP-2 in the early diagnosis of CI-AKI.

## 2. Materials and methods

### 2.1. Study design and participants

Patients were prospectively enrolled at the Children's Hospital of Hunan Province (Hunan, China) from May 2018 to January 2019. Eligible patients were between the ages of 0 and 18 years. Patients who received an intravascular injection of CM as a contrast agent were included. We excluded patients without detected SCr levels were at injection of the contrast agent, or within seven days before screening or within 48–72 h after injection, and patients with preexisting renal insufficiency and concomitant nephrotoxic drug use were excluded. There were 35 patients in the healthy control group. This clinical trial had been registered in Chinese Clinical Trial Regis (http://www.chictr.org.cn; Identifier: ChiCTR1800016913).

### 2.2. Data collection

Baseline demographic and clinical data, including age, gender, medical history, body mass index (BMI) and laboratory investigations, were recorded.

### 2.3. Definition of CI-AKI

CI-AKI is most commonly defined as an increase in SCr concentration of more than 44 µmol/L (0.5 mg/dL) or relative 25% increase in SCr levels at 48–72 h after exposure to contrast media in the absence of an alternative etiology [3].

### 2.4. Sample collection and measurement of biomarkers

Blood samples were obtained at the baseline (just prior to contrast injection) and at 48–72 h after contrast injection in the contrast agent group. SCr was detected by an automatic biochemistry analyzer (Shanghai Zhicheng Biological Technology Co., Ltd, Shanghai, China).

Serial urinary samples were obtained at baseline and at 2, 6, 12, and 24 h after intravascular injection of CM. Samples were immediately processed, aliquoted, and stored at below 20 °C until biomarker measurement without prior thaw. These samples were collected once in the healthy control group. Laboratory personnel performing biomarker analysis was blinded to clinical information about the participants. Urinary biomarker measurements were performed by enzyme-linked immunosorbent assay (Elabscience Biotechnology Co., Ltd, Wuhan, China). NGAL, IGFBP-7, and TIMP-2 levels are reported in units of ng/mL, [IGFBP-7]*[TIMP-2] levels are reported in units of (ng/mL)^2^/1000.

### 2.5. Statistical analysis

SPSS version 18.0 was used for all analyses. Continuous variables are reported as the median with interquartile range (IQR) due to a skewed distribution. Categorical variables are described as counts and percentages (%). Skewed distribution data were compared with the Wilcoxon rank sum test and Kruskal–Wallis test. To measure the sensitivity and specificity for urinary biomarkers for the prediction of CI-AKI, receiver operating characteristic (ROC) curves were generated, and the area under the curve (AUC) was calculated.

## 3. Results

### 3.1. Baseline characteristics and incidence of CI-AKI

We prospectively enrolled 172 children, all of whom had normal renal function before inclusion in the study. A total of 137 subjects were intravascularly injected with CM; 56.2% were male, and the baseline SCr was 24.00 (19.70–31.05) µmol/L. In 107 of 137 children who used iodixanol injection, the average contrast agent dose was 3 mL per kilogram. In the remaining 30 congenital heart disease patients who underwent elective cardiac catheterization and angiography and received an iopamidol injection, the average volume of contrast agents was 2 mL per kilogram but did not exceed 30 mL. The frequency of CI-AKI was 14.59%, based on the SCr outcome. We classified subjects into those with and without CI-AKI. At baseline, the patients in the CI-AKI group had significantly lower SCr level than their counterparts in the non-CI-AKI group. Baseline characteristics are shown in Table 1. Compared with the patients in the non-CI-AKI group, the patients in the CI-AKI group were not significantly different in age, BMI, proportion of males, hypoalbuminemia, proteinuria, fever, anemia, surgery, sepsis or abnormal liver function (*p* > 0.05). Median urinary baseline NGAL, IGFBP-7, and TIMP-2 concentrations were not significantly different between the CI-AKI group, non-CI-AKI group, and healthy controls (Table 1).

**Table 1.** Patient demographics and clinical outcomes.

	CI-AKI group	Non CI-AKI group	Healthy controls group	*p* Value
Cases (*n*)	20	117	35	
Gender (*n*, male/female)	13/7	64/53	19/16	0.678
Age (years)	1.76 (1.09–4.54)	3.2 (2.07–6.61)	2.20 (1.20–4.10)	0.058
BMI (kg/m^2^)	15.89 (14.2–18.76)	15.19 (14.42–16.89)	15.35 (13.41–16.51)	0.334
Clinical basics *n*(%)				
Thin	3 (15%)	18 (15.38%)	–	0.965
Obesity	3 (15%)	7 (5.98%)	–	0.152
Hypoalbuminemia	0	1 (0.85%)	–	0.687
Blood in the urinary	1 (5%)	14 (11.97%)	–	0.357
Proteinuria	1 (5%)	9 (8.41%)	–	0.669
Fever	5 (25%)	17 (15.88%)	–	0.119
Anemia	7 (35%)	26 (24.29%)	–	0.183
Surgery	16 (80%)	70 (59.82%)	–	0.085
Sepsis	2 (10%)	6 (5.13%)	–	0.282
Abnormal liver function	3 (15%)	9 (8.41%)	–	0.214
SCr				
Baseline µmol/L	18.25 (12.83–22.25)	24.20 (21.00–33.00)*	21.40 (17.90–27.80)*	0.005
48–72 h after contrast administration µmol/L	25.10 (19.25–32.83)	25.00 (18.90–31.80)	–	0.831
Baseline				
uNGAL ng/mL	27.69 (22.27–46.09)	27.37 (17.26–42.46)	23.49 (14.65–34.94)	0.315
uIGFBP-7 ng/mL	110.64 (78.53–156.84)	140.72 (112.50–161.66)	125.28 (113.61–141.91)	0.052
uTIMP-2 ng/mL	1.97 (1.55–2.75)	2.13 (1.46–2.51)	1.99 (1.82–2.34)	0.932
[uIGFBP-7]*[uTIMP-2] (ng/mL)^2^/1000	0.206 (0.186–0.294)	0.261 (0.202–0.359)	0.247 (0.199–0.321)	0.251

CI-AK: Contrast medium-induced acute kidney injury; BMI: Body Mass Index; uNGAL: neutrophil gelatinase-associated lipocalin; uIGFBP-7: insulin like growth factor binding protein-7; uTIMP-2: tissue inhibitor of metalloproteinase-2.

*p < 0.05 vs. CI-AKI group.

### 3.2. Changes in biological marker levels in the urine

Baseline (0 h) urinary NGAL, IGFBP-7, TIMP-2, and [IGFBP-7]*[TIMP-2] levels were consistently low and comparable in the CI-AKI group and the non-CI-AKI group (Table 2). In the CI-AKI group, the NGAL, IGFBP-7, TIMP-2, and [IGFBP-7]*[TIMP-2] levels in the urine were significantly increased than before the injection of CM at 2 h and 6 h, and remained at high levels at 12 h. Urinary NGAL and IGFBP-7 levels did not change significantly in the non-CI-AKI group at each of the six time points; however, the levels of urinary TIMP-2 and [IGFBP-7]*[TIMP-2] were slightly elevated at 2 h, but significantly lower than those in the CI-AKI group at the same time point and would be decreased to the precontrast level at 6 h. The analysis and comparison of biological marker levels in the urine showed that urinary NGAL, IGFBP-7, TIMP-2, and [IGFBP-7]*[TIMP-2] concentrations in the CI-AKI group after the CM injection increased faster than SCr.

**Table 2.** Comparison of the change of urinary biomarkers at time after injection of CM.

	CI-AKI group	Non CI-AKI group	*p* Value
uNGAL ng/mL			
Baseline	27.69 (22.27–46.09)	27.37 (17.26–42.46)	0.674
2 h	51.74 (30.41–97.81)**	28.98 (19.16–43.11)	0.002
6 h	45.33 (27.99–68.89)**	28.42 (19.84–44.35)	0.007
12 h	35.48 (25.59–53.89)*	29.72 (17.24–48.57)	0.114
24 h	33.97 (26.27–48.32)	27.49 (17.13–45.57)	0.216
48 h	28.11 (19.71–37.32)	26.39 (17.39–44.39)	0.756
*p* Value	<0.001	0.356	
uIGFBP-7 ng/mL			
Baseline	110.64 (78.53–156.84)	140.72 (112.50–161.66)	0.079
2 h	151.68 (126.92–183.52)**	141.09 (117.21–167.20)	0.128
6 h	197.34 (154.36–249.62)**	138.74 (112.11–162.78)	<0.001
12 h	140.86 (111.38–185.09)**	136.33 (105.72–164.29)	0.262
24 h	117.16 (97.51–161.56)	135.48 (111.13–161.15)	0.205
48 h	126.09 (100.98–173.53)	132.69 (108.49–153.89)	0.814
*p* Value	<0.001	0.117	
uTIMP-2 ng/mL			
Baseline	1.97 (1.55–2.75)	2.13 (1.46–2.51)	0.791
2 h	2.26 (1.90–4.03)**	2.28 (1.60–2.68))**	0.088
6 h	3.98 (2.49–5.59)**	2.15 (1.46–2.73)	<0.001
12 h	2.38 (1.68–2.81)	2.20 (1.55–2.61)	0.357
24 h	2.07 (1.66–2.54)	2.07 (1.36–2.54)	0.705
48 h	1.77 (1.39–2.83)	2.10 (1.39–2.57)	0.833
*p* Value	<0.001	0.035	
[uIGFBP-7]*[uTIMP-2] (ng/mL)^2^/1000			
Baseline	0.206 (0.186–0.294)	0.261 (0.202–0.359)	0.128
2 h	0.377 (0.301–0.539)**	0.287 (0.235–0.386)**	0.003
6 h	0.833 (0.458–1.321)**	0.259 (0.201–0.376)	<0.001
12 h	0.287 (0.222–0.456)**	0.266 (0.191–0.363)	0.207
24 h	0.226 (0.189–0.298)	0.252 (0.177–0.381)	0.661
48 h	0.228 (0.194–0.335)	0.254 (0.175–0.337)	0.951
*p* Value	<0.001	0.012	

uNGAL: Neutrophil gelatinase-associated lipocalin; uIGFBP-7: insulin like growth factor binding protein-7; uTIMP-2: tissue inhibitor of metalloproteinase-2.

*p < 0.05, **p < 0.01 vs. baseline.

### 3.3. Urinary NGAL, IGFBP-7, TIMP-2, and [IGFBP-7]*[TIMP-2] for the diagnosis of CI-AKI

The area under the ROC curve diagnosis CI-AKI of NGAL in the urine 2 h after injection of CM was 0.718 (95% CI: 0.575–0.860). The area under the ROC curve for IGFBP-7 and TIMP-2 in the blood 6 h after CM injection was 0.779 (95% CI: 0.658–0.901) and 0.779 (95% CI: 0.650–0.908), respectively. The area under the ROC curve diagnosis of CI-AKI of [IGFBP-7]*[TIMP-2] in the urine 6 h after CM injection was 0.811 (95% CI: 0.681–0.941) (Table 3). The [IGFBP-7]*[TIMP-2] was better than IGFBP-7 or TIMP-2 analyte alone at diagnosing children with CI-AKI early.

**Table 3.** Performance of uNGAL, uIGFBP-7, uTIMP-2, and [uIGFBP-7]*[uTIMP-2] for the diagnosis of CI-AKI.

	uNGAL	uIGFBP-7	uTIMP-2	[uIGFBP-7]* [uTIMP-2]
AUC	0.718	0.779	0.779	0.811
95% CI	0.575–0.860	0.658–0.901	0.650–0.908	0.681–0.941
Cutoff value	36.274	153.061	2.951	0.417
Sensitivity (%)	0.70	0.80	0.75	0.80
Specificity (%)	0.684	0.667	0.821	0.812

## 4. Discussion

Nephrotoxicity is frequently observed as a serious side effect in human patients after receiving iodinated contrast media, and contrast medium-induced acute kidney injury (CI-AKI) has been reported to be the third leading cause of acute kidney injury (AKI) in hospitalized patients [1]. CI-AKI is a serious but often overlooked complication. Most commonly, it is defined as either an absolute (0.5 mg/dL; 44 µmol/L) or relative 25% increase in SCr levels at 48–72 h after exposure to contrast media in the absence of an alternative etiology [3]. The pathogenesis of CI-AKI is very complicated and not fully understood. A reduction in renal perfusion caused by contrast media and direct toxic effects on tubular cells are generally accepted as the main factors in the pathophysiology of CI-AKI [4,14]. The incidence of CI-AKI has been reported to be approximately 11% [1], and we found that CI-AKI occurred in 14.59 (20/137) of the patients.

In our study, the proportion of patients with fever, anemia, surgery, sepsis and abnormal liver function tests in the CI-AKI group was higher than that of the non-CI-AKI group, worse health conditions found more common in CI-AKI group. Although the difference between the two groups was not statistically significant, we considered that the small sample size may cause limit the discovery of risk factors with CI-AKI.

Currently, the diagnosis of AKI relies on elevated SCr levels. However, SCr does not accurately depict kidney function and may not change until several days in patients with AKI. NGAL is a 25-kDa protein, a member of the lipocalin family. It is expressed at low concentrations in the normal kidney, trachea, lungs, stomach, and colon and is easily detected in the blood and urine soon after the kidney is damaged [9,10]. In a study of 91 children with congenital heart diseases, Hirsch et al. [8] defined CI-AKI as a 50% increase in SCr level from the baseline, which occurred in 11 participants (12%). They revealed that urinary NGAL was significantly increased at 2 h and 6 h after CI-AKI. Thus, NGAL might represent an early and sensitive biomarker for CI-AKI. In our study, we found that urinary NGAL was increased at 2 h and that it remained at a high level at 6 h in patients with CI-AKI, which was similar to that observed by Hirsch et al. [8].

Studies have indicated that IGFBP-7 and TIMP-2 are involved in G1 cell cycle arrest during the very early phase of cellular damage and impede the progression of cell division until DNA damage is repaired [13,15]. IGFBP-7 and TIMP-2 are protective molecules involved in cell apoptotic, inflammatory [16,17], and ischemic processes due to a variety of insults. Cuartero et al. [18] suggested that [IGFBP-7]*[TIMP-2] predicts AKI in both septic and nonseptic critically ill patients. Urinary [IGFBP-7]*[TIMP-2] levels were significantly higher in patients with AKI than in those without AKI. A multicenter prospective observational study of 94 infants, conducted by Gist et al. [19], demonstrated that urinary [IGFBP-7]*[TIMP-2] was significantly higher in patients with AKI. It can effectively predict the occurrence of AKI following cardiac surgery in infants [20].

In this study, we found that [IGFBP-7]*[TIMP-2] levels in urine were significantly increased in the CI-AKI group at 2 h and 6 h after CM injection, and the level of [IGFBP-7]*[TIMP-2] was significantly higher than that of the non-CI-AKI group at 6 h (Figure 1). The area under the ROC curve for IGFBP-7 and TIMP-2 in urine was 0.779 (95% CI: 0.658–0.901) and 0.779 (95% CI: 0.650–0.908), respectively, and the area under the ROC curve for [IGFBP-7]*[TIMP-2] was 0.811 (95% CI: 0.681–0.941). This proves that [IGFBP-7]*[TIMP-2] was better than IGFBP-7 or TIMP-2 analyte alone for the early diagnosis of CI-AKI in children. It was not anticipated that the levels of TIMP-2 and [IGFBP-7]*[TIMP-2] in urine would be slightly elevated at 2 h in the non-CI-AKI group, but significantly lower than those in the CI-AKI group at the same time point. It is highly possible that children without CI-AKI are likely to have transient, mild renal impairment after contrast injection.

The limitation is that our study is a small-scale study from a single center and did not identify the risk factors for CI-AKI. Our results need to be confirmed in other prospective multicenter studies with large sample sizes. For the treating clinician, it is often important to improve our diagnostic acumen to support early identification and treatment for patients with CI-AKI. To date, lack of early and sensitive biomarkers for children with CI-AKI has impaired our ability to intervene in a timely manner. A reliable early biomarkers that recognizes CI-AKI before SCr increases is still being sought. Our study shows that urinary NGAL, IGFBP-7, and TIMP-2 are sensitive, novel biomarkers used to diagnose CI-AKI.

## Data Availability

The raw data supporting the conclusions of this manuscript will be made available by the authors, the datasets during the current study are available in the website of http://www.chictr.org.cn/login.aspx?referurl=%2faddproject2.aspx Non CI-AKI group and CI-AKI group the level of uNGAL, uIGFBP-7, uTIMP-2, and [uIGFBP-7]*[uTIMP-2] at 2 h, 6 h.
